# Analysis of Risk Factors for First Seizure after Stroke in Chinese Patients

**DOI:** 10.1155/2013/702871

**Published:** 2013-11-02

**Authors:** Guoqing Wang, Haiyan Jia, Chunfu Chen, Senyang Lang, Xinfeng Liu, Cheng Xia, Yan Sun, Jun Zhang

**Affiliations:** ^1^Department of Neurology, Shandong Provincial Hospital Affiliated to Shandong University, Jinan, Shandong Province 250021, China; ^2^Department of Neurology, People's Hospital of Binzhou, Binzhou, Shandong Province 256610, China; ^3^Department of Neurology, Jinan Military General Hospital, Jinan, Shandong Province 250031, China; ^4^Department of Neurology, PLA General Hospital, Beijing 100853, China; ^5^Department of Neurology, Nanjing General Hospital of Nanjing Military Command, Nanjing 210002, China; ^6^Department of Neurology, Shenyang Military General Hospital, Shenyang 110015, China; ^7^Department of Cardiovascular Functional Studies, China Meitan General Hospital, Beijing 100028, China

## Abstract

The aim of this study is to assess related risk factors and predict early- and late-onset seizure after first-ever stroke. A total of 2474 consecutive patients with initial stroke in China from 1997 to 2007 were retrospectively investigated, in which, 24 clinical and radiological indexes were used for evaluation. Odds ratio (OR) and 95% confidence interval (CI) were calculated by logistic regression. A total of 232 (11.1%) of patients developed seizures during a mean follow-up period of 18 months, with 123 experiencing early-onset and 109 late-onset seizure. The independent risk factors for early-onset seizure were large lesion (OR = 9.36), subarachnoid hemorrhage (OR = 5.28), initial hyponatremia (OR = 2.10), and cortical involvement (OR = 1.33). The independent risk factors for late-onset seizure were cortical involvement (OR = 11.84) and large lesion (OR = 1.87). These results demonstrated that the risk factors for early seizure after stroke are large lesion, subarachnoid hemorrhage, and cortical involvement. Surprisingly, hyponatremia also predicts seizure in stroke patients. Cortical involvement is a major risk factor for late-onset seizure after stroke.

## 1. Introduction

In a large epidemiological study, cerebrovascular diseases represented the most commonly identified etiology of secondary epilepsy (11%) [1]. The role of stroke as an important cause of seizure, particularly in the elderly, has been accepted [2]. Early-onset seizures are common after stroke and have been reported to occur in 4.2% patients within 14 days after stroke [3]. 

Poststroke seizures are often characterized as “early” and “late” ones with definitions varying considerably [4–6]. Acute poststroke seizures are commonly classified as early and late ones based on differences in the presumed pathophysiology [7] and prognosis [8, 9], but there is no consensus on the differentiation between both seizures. In most studies, early seizures are defined as seizures occurring within 7–14 days after stroke symptom onset. The incidence of combined “early” and “late” seizures after stroke has been reported to be approximately 10% (5% of early seizures peaking within 24 h and 5% of late seizures predominantly within 6–12 months after a stroke) [10]. However, this varies significantly among studies due to many factors including differences in study design, stroke subtype(s) in final analysis, and duration of followup [4–6, 11]. Therefore, comparisons among studies are difficult.

The Oxfordshire community stroke project (OCSP), which examined the immediate and long term risk for seizures after first-ever stroke with a minimal follow-up period of two years in stroke survivors, reported that 11.5% of stroke patients were at a risk for developing post-stroke (i.e., delayed) seizures by five years [12]. It is known that seizure worsens the prognosis of stroke and complicates the acute stroke [8, 13]. Bogousslavsky et al. [14] described a persistent worsening of neurological outcome following epileptic seizures in stroke patients. 

In terms of poststroke seizures, most large studies focus on ischemic and hemorrhagic stroke or hemorrhagic stroke alone [12, 15–17]. Especially in China, there are 2 million patients suffering from a stroke annually, with the incidence of up to 120/10 million. Thus, to assess the risk factors for and predict the occurrence of seizures after first-ever stroke in China is much more important. There were two single-centered studies in China. One hospital-based cohort study from Hong Kang reported that seizures occurred in 3.4% of Chinese patients within one year after stroke onset, and 56% of early seizures were partial type whereas 72% of late seizures were generalized tonic-clonic type of undetermined onset. Only male gender (adjusted OR 3.21) and cortical location (adjusted OR 3.83) were significant independent risk factors, and intracerebral and subarachnoid haemorrhage (SAH) were not risk factors in this study [16]. Another study from Taiwan showed that the incidence of early seizure was 2.5% in Chinese inpatients with acute strokes. No correlation was found between seizure and stroke subtypes. The incidence of early seizure was significantly higher in acute stroke patients with cortical lesions than in those without cortical involvement [18]. 

Many studies examining the incidence of early poststroke seizures focus on comparing the characteristics and outcomes of early and late seizures [12, 15, 19–30]. However, the small sample size may significantly limit the identification of seizure predictors [30]. Thus, we conducted a multicentered study on stroke patients and multivariate analyses were employed to identify the risk factors for seizure after a stroke. 

## 2. Subjects

This multicentered study was undertaken in the Shandong Provincial Hospital Affiliated to Shandong University, People's Hospital of Binzhou, Jinan Military General Hospital, General Hospital of People Liberation Army, Nanjing General Hospital of Nanjing Military Command, Shenyang Military General Hospital, and China Meitan General Hospital. From November 1997 to September 2007, a total of 2474 consecutive patients with first-ever stroke were admitted to hospitals above mentioned within 48 h of symptom onset. Stroke was defined according to the World Health Organization criteria [31] as rapidly developed clinical signs of focal disturbance of cerebral function lasting for more than 24 h. The exclusion criteria were as follows: (1) patients with cardioembolic stroke [30] or cerebrovenous thrombosis [32]; (2) patients without available previous hospital records; (3) patients with impractical followup or a new acute event of stroke; (4) patients with a history of seizure or epilepsy before admission; (5) patients with status epilepticus including nonconvulsive or convulsive status epilepticus [30]; (6) patients who died before epileptic seizure and patients with causes other than vascular origin, such as a history of severe head trauma, brain tumor, brain surgery, and central nervous system infections; (7) the loss of consciousness or short-lasting episodes of mental confusion; (8) acute agitated confusional state [33], defined on the basis of criteria for delirium described in the Diagnostic and Statistical Manual of Mental Disorders [34], diagnosed according to medical history, initial examinations, and observations within 48 h of hospitalization; (9) other common causes of confusion in the elderly (intoxications, dysmetabolic diseases, sepsis, and withdrawal state) [5]; (10) limb-shaking transient ischemic attack (TIA) and decerebrate posturing; (11) silent strokes confirmed by cranial computed tomography (CT) or magnetic resonance imaging (MRI). A total of 2094 (84.6%) patients with first-ever stroke met the inclusion criteria. Demographic characteristics, clinical information and findings from neurological examination, laboratory tests (complete blood cell count, routine blood biochemical examination, serum electrolyte examination, and urinalysis), chest roentgenography, and 12-lead electrocardiograph were collected on admission. All patients underwent the cranial CT scan on admission, which was done again within the first week of stroke onset in patients with negative results in CT scan (*n* = 364, 14.7%). Cerebral MRI was performed in selected patients (e.g, patients with negative results in a second CT scan (*n* = 47, 1.9%)). Electroencephalography (EEG) was performed within 24–48 h after seizure onset in all patients. EEG electrodes were placed according to the International 10–20 system. Activation procedures such as hyperventilation and photic stimulation were employed. In addition to cranial CT, lumbar puncture was also done to confirm SAH. Seizures were diagnosed and classified according to the criteria developed by the International League Against Epilepsy [35]. The diagnosis was based on the direct observation of seizures by medical staff at the time of hospitalization, neurologists' report of seizure, and reliable descriptions of seizure by personnel in ambulance during transportation or from patients or their relatives when seizures occurred at home. Based on seizure onset in relation to the clinical ictus of cerebrovascular disorders, patients with seizures were slightly modified from those of previous studies [31]. Early-onset seizure was defined as a seizure occurring within 14 days after stroke and late-onset seizure as a seizure occurring more than 14 days after stroke. The medical records since the stroke were reviewed in all patients. Survivors were followed up by telephone or interviewing. If necessary, their relatives, family physicians, or staff in hospital or nursing home was consulted. Followup was carried out for a mean period of 18 months (range: 0.5–2 years) and terminated by the end of the study (September 2007). Written informed consent was obtained from all patients. The study was approved by the Institutional Review Committee.

## 3. Methods

Information was collected by using a structured questionnaire including the age, sex, history of hospitalization, family history, types of initial stroke, location of lesion, size of lesion, symptoms and concomitant diseases at seizure onset, types of seizure, time of seizure after stroke, disabling deficits (National Institutes of Health Stroke Scale (NIHSS)) on admission, medications used, events preceding the stroke onset, and past medical history. Previous hospital records were available and were checked for seizure and nonseizure patients. All the seizure patients were interviewed by the same physician in each center. For seizure patients who were confused, unconscious, dysphasic, or who died soon after admission, family members were personally interviewed. Some of the nonseizure control subjects were followed up by telephone or interviewing. All these information was input in a specially designed proforma.

The types of lesion were defined according to previously described criteria [17]. Lesion size was determined by the largest diameter of lesions on CT images. Lesions were classified as small (≤35 mm in diameter and <5 slices) and large lesions (>35 mm in diameter and ≥5 slices). The “cortical” lesion referred to one confined to the cortical or corticosubcortical (total or partial involvement of supratentorial cerebral cortex) regions. The “subcortical” lesion referred to one locating exclusively beneath the cerebral cortex. Hypertension was confirmed once patients were treated with antihypertensive drugs on admission or hypertension was diagnosed during the hospital stay. Diabetes mellitus was defined on the basis of medical history of diabetes or diagnosed during the hospital stay. Smokers were categorized into current smokers and ex-smokers who quit smoking more than one year ago. Heavy drinking was referred to as a mean daily alcohol intake exceeding 60 g of ethanol for more than one year preceding the stroke onset.

## 4. Statistical Analysis

Statistical analysis was performed with SPSS version 10.0 for Windows. In univariate analysis, independent-sample *t*-test was used for age comparison and chi-square test was employed for comparisons of noncontinuous variables. Variables were subjected to multivariate analysis with a logistic regression and forward stepwise selection after univariate analysis. The presence or absence of seizure (coded as 0 or 1, resp.) was the independent variable. An unconditional multiple logistic regression model was used to calculate odds ratios (OR) and 95% confidence interval (CI). A value of *P* < 0.05 was considered statistically significant.

## 5. Results

In 2094 inpatients with first-ever stroke, the age ranged from 32 to 92 years, there were 1393 men and 701 women, and 232 (11.1%) developed seizure. In 232 patients with poststroke seizure, the age ranged from 32 to 92 years, there were 153 men and 79 women, 96 (41.4%) had cerebral infarction, 92 (39.7%) cerebral hemorrhage, 5 (2.2%) hemorrhagic infarction, 4 (1.7%) cerebral hemorrhage with concomitant cerebral infarction, and 35 (15.1%) SAH. Of 232 seizure patients, 123 (mean age: 62.8 years: range, 32 to 92 years; 81 men) experienced early-onset seizure (early seizure group) and 109 (mean age: 64.5 years; range: 37 to 89 years; 73 men) developed late-onset seizure (late seizure group). The remaining 1862 patients (nonseizure group) (mean age: 57.2 years; range: 35 to 88 years; 1240 men) had the following cerebrovascular accidents: 830 (44.6%) had cerebral infarction, 778 (41.8%) cerebral hemorrhage, 29 (1.6%) hemorrhagic infarction, 17 (0.9%) cerebral infarction and concomitant cerebral hemorrhage, and 208 (11.2%) SAH. The characteristics of all patients at baseline are listed in Table 1. As for seizure subtypes, of 232 patients with poststroke seizure, 77 patients (33.2%) had simple partial seizures, 80 (34.5%) secondary generalized tonic-clonic seizures, and 75 (32.3%) complex partial and tonic-clonic seizures.

 All the 232 patients presented with abnormal EEGs. Diffuse slowing of background activity was the most common (67/232, 28.9%). Focal slowing and epileptiform activity were noted in 50/232 (21.5%) and 115/232 (49.6%), respectively. In these 115 patients, the abnormalities included ictal activity in 20 (17.4%), focal sharp and slow waves in 35 (30.4%), focal spikes and slow waves in 20 (17.4%), focal sharp waves in 18 (15.7%), focal spike waves in 14 (12.2%), and periodic lateralized epileptiform discharges (PLEDs) in 8 (7.0%). PLEDs were not found in nonseizure patients. Many studies have reported that PLEDs were associated with acute stroke and subsequent development of seizures [36]. In our cohort, PLEDs were seen in 7.0% of stroke patients, which was consistent with what was previously reported by De Reuck et al. (5.8%) [37] and Mecarelli et al. (6%) [38]. Of 232 patients, lesions were found in multiple sites, 92 in 232 patients (39.7%), 99 in the cortical area (42.7%), one in the cerebellum (0.4%), 40 in the subcortical area (17.2%), and 0 in the brain stem.

Patients in nonseizure group were younger than those in early seizure group (*t* = 3.394, *P* < 0.01) and late seizure group (*t* = 4.187, *P* < 0.01). NIHSS score was 10.3 ± 10.1, 12.1 ± 9.5, and 12.2 ± 10.3 in nonseizure group, early seizure group, and late seizure group, respectively, showing no significant difference between nonseizure group and early seizure group (*t* = 1.921, *P* = 0.055) or late seizure group (*t* = 1.907, *P* = 0.057). Other potential risk factors and results of univariate analysis are shown in Table 2. Significant differences between patients with and without early seizures were found in SAH, cortical lesion, multiple sites, large size of lesion, fever, infection, kidney dysfunction, upper digestive tract hemorrhage, electrolyte disturbance, and age. Significant differences between patients with and without late seizures were found in cortical lesion, multiple sites, and large size of lesion, hypertension and age.

Results of multiple logistic regression analysis are shown in Table 3. Age was used as a continuous variable with a constant OR for each year. Multivariate analysis showed that only large lesion (OR = 9.36), SAH (OR = 5.28), hyponatremia (OR = 2.10), and cortical involvement (OR = 1.33) appeared to be independent risk factors for early seizures after first-ever stroke. On multivariate analysis, cortical involvement and large lesion remained significant predictors of late-onset seizures. In order to determine the independent influence of factors above, dichotomous variables were coded as 0 or 1.

## 6. Discussion 

Seizure is one of the most common neurological disorders in the elderly. In this population, stroke is the most commonly identified cause of seizure. Three studies reported the rates of poststroke seizure as 9% [39], 14% [40], and 19% [13], respectively. In a hospital-based cohort of 1000 Chinese patients, Cheung et al. reported that 3.4% of patients developed seizures within one year after stroke [16]. Our results showed that seizure occurred in 11.1% in stroke patients admitted to our departments. Some authors have proposed that immediate or early seizures are associated with a significant risk for recurrence [25]; others have observed that early seizures do not predict late seizures [41]. The reported recurrence rates vary from 28% to 93%, depending on whether immediate, early, or late seizures are considered and whether repetitive seizures or clusters of seizures are considered as a single seizure episode or as recurrent seizures [15, 23, 25, 39, 42]. In our study, the relapse rate was not clear since patients with repetitive seizures were excluded.

Some, but not all, previous studies have indicated that there is a relationship between lesion size and poststroke seizure, which was confirmed in our study. In multivariate analysis, the rate of early seizure was 9.36-fold higher in stroke patients with large lesion than that in those without large lesion. Lesion size exclusively reflects the stroke severity. The relationship between stroke severity and seizures is still controversial [3, 8, 15, 43]. Reith et al. [3], Burn et al. [12], and Bladin et al. [15] found that the stroke severity was the unique predictor of early seizure. However, Gupta et al. [44] found that NIHSS score was not a predictor for seizures after first ischemic stroke and only the presence of ischemic cortical infarction (*P* = 0.009, OR = 5.549, 95% CI = 1.53–20.19) was independently associated with seizures. In the present study, the severity of stroke patients with seizures was not higher. It has been reported that there was no significant difference in overall lesion size between patients with and without seizures [15]. On their multivariate analysis, only cortical lesion (not hemorrhage size, disability, and cerebrospinal fluid blood) was identified as a risk factor for seizures after stroke including cerebral infarction and ICH. A relationship between seizure and cortical involvement has been reported in many studies except for study of Reith et al. [3]. The only risk factor consistently found to be a risk factor of seizure is cortical location, particularly in ischemic stroke [9, 12, 15, 18, 45]. Our findings showed a significant association between cortical involvement and early seizures in both univariate and multivariate analyses. The association was even stronger (OR = 11.84, *P* < 0.01) when the analyses were confined to late-onset seizures, suggesting that cortical lesions are of a particular relevance for later-onset seizure after a stroke. In univariate analysis, hypertension was found to be associated with late seizures, which were not observed in the multivariate analysis, suggesting that hypertension is a confounder for late seizures. Surprisingly, hyponatremia was a risk factor for early post-stroke seizure, independent of other risk factors. This may be explained in part by the cellular biochemical dysfunction [15]. Age did not appear to be an independent predictor of seizures in multivariate analysis in the present study, which was consistent with a prior study [12]. Age is believed to be a confounding factor for poststroke seizure. 

Seizures are an important neurological complication of spontaneous ICH. In a 1-year retrospective Chinese study in which 243 adult patients were enrolled, investigators found that the mean ICH volume was independently associated with seizures [46]. Some investigators have reported an association between seizures and large lesions, which is based on analysis of patients with ischemic and hemorrhagic stroke [3, 17, 24, 47], as in the present study. However, this association was not found in patients with ICH [48] or patients with seizures had smaller hemorrhages than those without seizures [41, 49]. Lobar lesion has been widely recognized as the most potent predictor of immediate seizures [18, 25, 28, 42–44, 46, 50]. Lobar hemorrhage continues to be an independent predictor of early seizures, indicating that patients with lobar ICH are susceptible to seizures, but neurologic complications also contribute significantly to the occurrence of early seizures. Faught et al. [41] followed up 123 patients with primary ICH, defined as bleeding without known precipitating cause except for hypertension, for an average of 4.6 years or until death in order to determine the incidence, prevalence, and type of epileptic seizures. Their results showed 25% of patients developed seizures. In 50% of these patients, seizure incidence was higher in patients with lobar hemorrhage (54%), lower in patients with basal ganglionic hemorrhage (19%), and zero in patients with thalamic hemorrhage. The temporal or parietal involvement predicted poststroke seizures. Berger et al. [48] found seizures in 19 of 112 patients (17%) with nontraumatic, supratentorial ICH. Hemorrhage involving the cerebral cortex, regardless of site of origin, predisposes to seizures. The 30-day actuarial risk for a post-ICH seizure was 8.1%. Lobar lesion and ICH small volume were independent predictors of immediate (within 24 h of ICH) seizures. Early (within 30 days of ICH) seizures were associated with lobar lesion and neurologic complications, mainly rebleeding [42]. Our findings were compared with those reported in the previous literatures in order to highlight main factors affecting the occurrence and development of seizures after spontaneous ICH. In our study, the cerebral hemorrhages giving rise to seizures were predominantly cortical in location, similar to previous studies [26, 48, 49]. Early post-ICH seizures were associated with lobar lesion and neurologic complications, mainly rebleeding. Rebleeding as a prognostic factor for early seizures has occasionally been reported in studies on patients with ICH, whereas it is a major predictor of seizures in patients with SAH [43, 50]. 

Some authors have emphasized the early prophylactic antiepileptic treatment in SAH due to rupture of an intracranial aneurysm. In agreement with observations by Hasan et al. [51] and Arboix et al. [17], our findings revealed that SAH was an independent predictor of early seizure in stroke patients. A key question is whether late seizures are associated with SAH as little is known about the predictive value of first SAH on late-onset seizures. Previous studies on late seizures after SAH had a small sample size, and multivariate analyses were not used. In a previous study, it was found that late seizures within the first six weeks were independently related to subarachnoid rebleeding (OR = 94, *P* < 0.01) and seizures at stroke onset (OR = 27, *P* < 0.01), but not to other variables related to stroke onset, development of hydrocephalus, and vasospasm [13]. However, subarachnoid rebleeding patients were not included in this study. Our study, for the first time, showed that first-ever SAH was not a significant risk factor for late seizures. This, however, should be explained with caution, as the number of SAH patients was small in our study.

There were following findings in this study. First, the incidence of seizure in patients with stroke was 11.1%. Second, on the basis of multivariate analysis, there was no relationship between seizure and risk factors for stroke (such as hypertension, diabetes mellitus, and coronary heart disease). Third, the predictors of early seizure are lesion size, SAH, and cortical involvement. In addition, hyponatremia at stroke onset is an independent predictor of poststroke seizure. Fourth, late seizures are mainly associated with cortical involvement. It is suggested that the role of anticonvulsant drugs in prophylaxis of seizures should be assessed in prospective, randomized, double-blind clinical trials on patients with high risk for poststroke seizures. 

This study still has several limitations. First, this is a retrospective study and therefore subject to the influence of unmeasured factors. Attention to careful interviewing of all the subjects is the strength of our study. However, recall bias by patients and their relatives is possible because some of them were interviewed via telephone without informing them about upcoming interviews. We tried to minimize this bias via conducting interviews by the same person according to a structured protocol and carefully reviewing previous hospital records of all the subjects. Second, it is impossible to assess the effect of prophylactic antiepilepsy drugs after acute stroke in the prevention of subsequent seizures. Third, patients with preexisting neurologic deficits have been excluded. Thus, there is uncertainty in assessing the incidence of seizures after stroke in nonselected patients. Fourth, the number of patients was too small to perform a multivariate analysis of risk factors with regard to type and severity of seizures in the present study. Fifth, the risk for seizure recurrence in patients with early seizures was not investigated. Therefore, randomized, prospective, multicentered studies with large sample size are required to confirm our findings.

## Figures and Tables

**Table 1:** Characteristics of patients with poststroke seizure at baseline.

Characteristics	Early seizure (*n* = 123)		Late seizure (*n* = 109)	
	*n*	%	*n*	%
Signs and symptoms				
Headache	17	(13.8)	11	(10.1)
Dizziness	11	(8.9)	3	(2.8)
Nausea, vomiting	9	(7.3)	0	(0)
Altered consciousness	21	(17.1)	0	(0)
Speech deficits	36	(29.3)	15	(13.8)
Cranial nerve palsy	89	(72.4)	57	(52.3)
Limb paresis	89	(72.4)	82	(75.2)
Hemianopia	5	(4.1)	1	(0.9)
Ataxia	18	(14.6)	8	(7.3)
Hemihypesthesia	67	(54.5)	62	(56.9)
EEG				
Diffuse slowing	51	(41.5)	16	(14.7)
Focal slowing	21	(17.1)	29	(26.6)
Epileptiform activity				
Ictal activity	11	(8.9)	9	(8.3)
Focal sharp and slow waves	13	(10.6)	22	(20.2)
Focal spikes & slow waves	18	(14.6)	2	(1.8)
Focal sharp waves	5	(4.1)	13	(11.9)
Focal spike waves	3	(2.4)	11	(10.1)
PLEDs	1	(0.8)	7	(6.4)
Seizure type				
Secondarily generalized tonic-clonic seizures	43	(35.0)	37	(34.0)
Simple partial seizures	23	(18.7)	54	(49.5)
Complex partial & tonic-clonic seizures	57	(46.3)	18	(16.5)
Stroke types				
Cerebral infarction	48	(39.0%)	48	(44.0%)
Cerebral hemorrhage	45	(36.6%)	47	(43.1%)
Hemorrhagic infarction	2	(1.6%)	3	(2.8%)
Cerebral hemorrhage with cerebral infarction	2	(1.6%)	2	(1.8%)
Subarachnoid hemorrhage	26	(21.1%)	9	(8.3%)
Lesion location on CT				
Cortical area	48	(39.0)	51	(46.8)
Subcortical area	26	(21.1)	14	(12.8)
Cerebellum	0	(0)	1	(0.9)
Brain stem	0	(0)	0	(0)
Multiple sites	49	(39.9)	43	(39.5)
Lobe of lesion*				
Frontal	41	(33.3)	33	(30.5)
Parietal	38	(30.9)	35	(32.4)
Occipital	15	(12.2)	12	(11.1)
Temporal	29	(23.6)	28	(25.9)

PLEDs: periodic lateralized epileptiform discharges. *One patient with late seizure had cerebellar stroke.

**Table 2:** Results of univariate analysis.

Variables	Early seizure		Late seizure	
	*χ* ^2^	*P*	*χ* ^2^	*P*
Gender	0.125	0.724	0.007	0.935
Family history of EP	0.331	1.000*	1.429	0.289*
Risk factors of stroke				
Family history of stroke	0.533	0.465	1.010	0.315
Alcohol abuse	0.063	0.801	0.186	0.666
Smoking	0.421	0.517	0.027	0.871
Comorbidity				
Diabetes mellitus	0.351	0.553	0.020	0.886
Hypertension	0.142	0.706	6.092	0.014
Coronary heart disease	0.004	0.950	0.001	0.970
Kidney dysfunction	6.753	0.009	0.118	0.731
Hypohepatia	2.067	0.151	2.931	0.087
Emphysema	1.678	0.372*	0.329	0.543*
Dyslipidaemia				
Stroke types				
Cerebral infarction	0.453	0.501	0.452	0.501
Cerebral hemorrhage	0.914	0.339	0.007	0.931
Hemorrhagic infarction	0.565	0.445*	0.051	0.688*
Cerebral hemorrhage with cerebral infarction	0.013	1.000*	3.468	0.095*
Subarachnoid hemorrhage	5.079	0.024	0.059	0.808
Stroke location				
Cortical area	6.903	0.003	20.720	<0.001
Subcortical area	3.204	0.073	17.201	<0.001
Cerebellum	13.099	<0.001*	9.435	0.002**
Multiple sites	6.295	0.032	8.426	0.044
Lobe of lesion				
Frontal	0.830	0.362	2.070	0.150
Parietal	0.167	0.683	0.519	0.470
Occipital	0.197	0.657	0.041	0.840
Temporal	0.076	0.782	0.682	0.409
Large lesion	5.517	0.023	16.314	<0.001
Complications after stroke				
Fever	15.165	<0.001	2.568	0.121*
Acid-base disturbance	2.034	0.154	0.691	0.404*
Electrolyte disturbance	9.386	0.002	3.637	0.056
Upper alimentary tract hemorrhage	8.974	0.003	0.444	1.000*
Infection	13.958	<0.001	1.639	0.201*
Depression	1.855	0.173	0.808	0.369

*Fisher's exact test. **Continuity correction.

Other complications after stroke included shoulder pain, deep venous thrombosis, and pulmonary embolism.

**Table 3:** Results of multivariate analysis.

Variables	*β*-coefficients	Standard errors	Standard *β*-coefficients	OR	95% CI	*P*
	Early seizure					
Large lesion	3.34640	0.52372	3.49463	9.36	2.84~16.68	0.001
Subarachnoid hemorrhage	2.24179	0.50025	2.58235	5.28	2.01~9.37	0.012
Electrolyte disturbance	0.59862	0.21274	1.27597	2.10	1.19~4.92	0.029
Cortical involvement	0.29887	0.12337	0.82648	1.33	1.02~2.36	0.041
Constant	−5.37482	1.39618	−5.29853			

	Late seizure					
Cortical involvement	4.32488	0.67751	4.52376	11.84	3.77~19.83	0.001
Large lesion	0.95039	0.29528	1.44647	1.87	1.08~2.96	0.039
Constant	−5.87396	1.43074	−5.06541			
